# Trajectories of Eating Behavior and Health-Related Quality of Life During the First Year After Metabolic Bariatric Surgery: A Longitudinal Study

**DOI:** 10.3390/healthcare14091198

**Published:** 2026-04-29

**Authors:** Shu Fen Wu, Hong Yi Tung, Yu Rong Hsu, Shih Ting Lo, Tien Chou Soong

**Affiliations:** 1 School of Nursing, College of Nursing, Kaohsiung Medical University, Kaohsiung 807378, Taiwan; y355400@gmail.com; 2 Department of Nursing, Yuh-Ing Junior College of Health Care & Management, Kaohsiung 807056, Taiwan; 3 Department of General Surgery, Yuan’s General Hospital, Kaohsiung 802635, Taiwan; tong0905@ms4.hinet.net; 4 Department of Weight Loss & Health Management Center, E-DA Dachang Hospital, Kaohsiung 807066, Taiwan; yurong.hsu0318@gmail.com; 5 Department of Nursing, E-DA Hospital, Kaohsiung 824005, Taiwan; 6 Department of Medicine, I-Shou University, Kaohsiung 824005, Taiwan

**Keywords:** metabolic bariatric surgery, eating behavior, health-related quality of life, group-based trajectory modeling, longitudinal study

## Abstract

Background: Metabolic bariatric surgery (MBS) yields significant but heterogeneous recovery patterns. The longitudinal interplay between evolving eating behaviors and health-related quality of life (HRQoL) remains insufficiently characterized. Objectives: To identify trajectories of eating behavior and HRQoL during the first postoperative year and examine their associations with 12-month outcomes. Methods: A total of 244 patients from two hospitals in Taiwan were followed for 12 months. Dutch Eating Behavior Questionnaire, and Impact of Weight on Quality of Life-Lite were assessed. Group-based trajectory modeling (GBTM) identified latent subgroups, and multiple regression analyzed associations with 12-month HRQoL, adjusting for clinical covariates. Results: GBTM identified two distinct trajectories for restrained, emotional, and external eating. For HRQoL, three trajectories emerged: high-start stable (45–50%), moderate-decline (30–35%), and low-start improving (~20%). In the regression model (R^2^ = 0.37, *p* < 0.001), eating behavior trajectories were not independently associated with total HRQoL at 12 months after adjusting for covariates, including baseline BMI and comorbidities. Specifically, restrained eating (β = −1.42, *p* = 0.502), emotional eating (β = −10.33, *p* = 0.110), and external eating (β = −5.33, *p* = 0.160) trajectories did not significantly predict global HRQoL scores. Conclusions: Postoperative adaptation is characterized by substantial heterogeneity, with a significant subgroup experiencing HRQoL decline despite surgery. While eating behavior trajectories align with domain-specific psychosocial trends, early postoperative clinical factors appear to exert a more dominant influence on total HRQoL during the first year. These findings suggest that multidisciplinary support should target specific vulnerable trajectories to optimize long-term outcomes.

## 1. Introduction

The global prevalence of obesity has risen markedly in recent decades, emerging as a critical public health challenge. Obesity is a multifactorial chronic disease that adversely affects psychological, and social well-being [1] and is a major risk factor for cardiovascular disease, type 2 diabetes, osteoarthritis, certain cancers, and premature mortality [2]. Metabolic bariatric surgery (MBS) remains the most effective and durable treatment for morbid obesity [3], achieving substantial and sustained weight loss while also improving comorbidities, social functioning, overall quality of life (QoL), and reduce mortality risk [4,5]. However, postoperative outcomes are not uniform across patients. Increasing evidence suggests considerable inter-individual variability in postoperative physical, behavioral, and psychosocial adaptation. Part of this variability may be attributable to differences in surgical procedures, as distinct bariatric techniques involve different restrictive, hormonal, and metabolic mechanisms that may differentially influence weight loss, eating behaviors, and HRQoL outcomes. Previous studies have reported procedure-specific differences in long-term bariatric outcomes and health-related quality of life across Roux-en-Y gastric bypass, one anastomosis gastric bypass, and sleeve gastrectomy [6,7]. Such heterogeneity may be overlooked by conventional mean-based analyses but can be more effectively captured through Group-Based Trajectory Modeling (GBTM), which enables the identification of clinically meaningful subgroups with distinct recovery patterns. Although MBS induces marked weight reduction, the maintenance of these benefits is closely linked to postoperative changes in eating behaviors, which also play a significant role in shaping QoL [8,9]. The first postoperative year represents a particularly critical period, characterized by rapid weight loss, major physiological adaptation, restructuring of eating habits, and psychological adjustment to changes in body image and lifestyle demands [10,11]. These concurrent transitions may disrupt pre-existing behavioral and psychological patterns, requiring continuous adaptation and leading to divergent recovery trajectories across individuals. Accordingly, this period provides a crucial window for understanding the dynamic evolution of eating behaviors and HRQoL following MBS.

Eating behavior is a key determinant of postoperative weight trajectories. Eating behavior encompasses the psychological, physiological, and environmental factors influencing food intake [9]. Individuals with morbid obesity often exhibit dysregulated dietary patterns such as irregular meal timing, heightened hunger, specific food preferences, along with frequent intake of high-calorie foods, sugar-sweetened beverages, and emotional eating etc, can undermine surgical benefits [12,13]. Eating Behavior Patterns, including emotional, external, and restrained eating, have been associated with poorer weight-loss outcomes, though findings are inconsistent: some studies show emotional eating negatively predicts weight loss, while others find no effect or only preoperative influence [14,15,16]. These inconsistencies underscore the need for longitudinal research to clarify how eating behaviors evolve post-MBS and their role in long-term weight-loss maintenance. In addition, by understanding how patients adapt to new eating habits, control emotional diet and cope with physical changes, they can provide valuable information about psychological and physical challenges after surgery, thus improving their QoL.

Health-related QOL (HRQoL) is a multidimensional construct reflecting the impact of illness and treatment on physical, psychological, and social well-being [17]. Individuals with obesity frequently experience social stigma, depression, anxiety, eating disorders, and body image disturbance, all of which impair HRQoL [18,19]. Improving the HRQoL is often a motivator for seeking bariatric surgery [20], and previous studies have shown greater improvements in physical than mental health domains, with maximal benefits observed 1–2 years postoperatively [21]. A systematic review revealed that while physical HRQoL gains may persist, psychological predispositions can limit long-term benefits in both HRQoL and weight reduction [22].

Existing cross-sectional and longitudinal evidence indicates that sociodemographic, psychosocial, and biomedical factors shape HRQoL trajectories after MBS [23,24]. Since the development of healthy eating habits is essential for sustained weight loss, and postoperative changes in dietary behavior can substantially influence patients’ QoL, both dietary behavior and HRQoL should be viewed as dynamic constructs that may evolve over time. Therefore, the aims of this study were to identify distinct postoperative trajectories of eating behaviors and HRQoL during the first year after MBS and to examine their interrelationships and associated factors, including their associations with HRQoL outcomes at 12 months. Based on existing evidence, we tested the following hypotheses: (1) multiple distinct trajectories would emerge for both eating behaviors and HRQoL; (2) maladaptive eating behavior trajectories particularly persistent emotional eating would be associated with poorer HRQoL outcomes; (3) baseline sociodemographic and clinical characteristics would be associated with trajectory patterns.

## 2. Materials and Methods

### 2.1. Study Design and Procedure

The prospective, longitudinal design was used in this study. The data were collected from January 2022 to December 2022. Participants in this cohort completed the questionnaires at baseline (1 wk), 1, 3, 6, 9 and 12 months by follow-up interviews at outpatient department. Data were collected through face-to-face questionnaire interviews conducted one week after surgery. Eligible participants were identified via the outpatient medical system, with attending physicians assisting in screening individuals who met the inclusion criteria. The researcher provided a detailed explanation of the study’s objectives, procedures, potential risks, and benefits. Participants who demonstrated full understanding were invited to provide written informed consent before completing the questionnaire. Upon completion, participants deposited their self-administered questionnaires into a designated collection box.

### 2.2. Clinical Care Context and Psychological Support

Preoperative evaluation was conducted as part of routine surgical assessment; however, a standardized psychological assessment protocol was not uniformly implemented. Postoperative care followed a standard clinical pathway. During follow-up, patients who exhibited psychological distress or maladaptive behavioral changes were managed according to clinical judgment, with referral to psychological counseling or psychiatric services when deemed necessary. These referrals were not based on predefined criteria but were provided on an ad-hoc basis at the discretion of the treating clinicians.

Although psychological support services were available, they were not systematically integrated into routine follow-up. Therefore, the observed trajectories should be interpreted within the context of routine clinical care, reflecting the natural course of postoperative adaptation rather than outcomes influenced by structured psychological interventions.

### 2.3. Sample Size Calculation

There is currently no consensus regarding the minimum sample size required for latent class growth analysis (LCGA) or group-based trajectory modeling (GBTM) [25]. However, prior methodological studies suggest that a sample size of at least 200 participants is generally sufficient to ensure stable estimation of trajectory models and reliable classification [26]. In the present study, sample size was calculated using G*Power (version 3.1.9.2). The minimum sample size was estimated as 208 for logistic regression analysis. The parameters were set as: two tailed test, odds ratio at 1.5, effect size index at 0.5, an α level of 0.05, power at 0.8, R^2^ value of 0, normal distribution, a minimum of 208 participants was required. Considering an anticipated attrition rate of 40%, which is consistent with recent longitudinal bariatric surgery studies reporting variable but often substantial loss to follow-up [11,27,28], the sample size was inflated and 300 eligible participants were recruited. Ultimately, 244 participants completed the study (completion rate: 81.3%), exceeding the minimum required sample size.

### 2.4. Participants

The participants of present study were recruited from two outpatient departments in Southern Taiwan. The general inclusion criteria were age of >20 years, for bariatric surgery when their BMI ≥ 40 kg/m^2^ or when their BMI is >35 kg/m^2^ with associated health problems (i.e., type 2 diabetes, hypertension, dyslipidemia or obstructive sleep apnea syndrome), and ability to communication in Mandarin Chinese. Exclusion criteria included prior bariatric or upper gastrointestinal surgery; clinically significant kidney disease (e.g., chronic kidney disease stage ≥3 or eGFR < 60 mL/min/1.73 m^2^, dialysis, or transplantation); and clinically significant liver disease (e.g., cirrhosis, decompensated liver disease, active hepatitis or cholestasis, or persistent marked elevation of liver enzymes). Patients with mild or stable non-alcoholic fatty liver disease without advanced fibrosis were not excluded. Severe psychiatric disorders, unstable psychological conditions, or active substance abuse that could impair adherence to postoperative care were also excluded, while stable, well-controlled conditions were permitted. Preoperative clinical evaluation was conducted as part of routine surgical assessment; however, a standardized psychological assessment protocol was not uniformly implemented for all patients.

### 2.5. Eating Behavior Assessment

Eating behavior traits were assessed with the support of the Dutch Eating Behavior Questionnaire (DEBQ). The scale consists of 33 items and has three subscales including (1) emotional eating behavior, that is, eating in response to emotional cues or to regulate emotion; (2) external eating behavior, that is, eating in response to food-related stimuli such as taste, smell, irrespective of hunger and satiety; and (3) restrained eating behavior, that is, dietary control via cognitive cues to influence one’s body weight. Each item is rated on a 5-point Likert-type scale ranging from 1 to 5, with higher scale scores indicating a stronger tendency towards that behavior. The total score ranges of the scale ranges from 33 to 165 [29]. The Chinese version validity and reliability studies of the scale were conducted by Wang et al. and the Cronbach’s alpha coefficients of the subscales were 0.81 to 0.94 [30]. In present study, the Cronbach’s alpha for the scale’s subscales were 0.86 to 0.94 and equaling 0.96 for the total score.

### 2.6. Health-Related Quality of Life Assessment

To assess HRQoL, the questionnaire of the Impact of Weight on Quality of Life-Lite (IWQOL-Lite) was used. The IWQOL Lite is a 31-item self-assessment questionnaire with five domains including physical function (11 items), self-esteem (7 items), sexual life (4 items), public distress (5 items), and work life function are the subjects addressed (4 items). Each item is rated on a 5-point Likert-type scale ranging from 1 to 5. The total score and all domain scores ranged from 0 to 100, with higher scores representing better weight-related QoL. The Cronbach’s alpha coefficients of the subscales were 0.90 to 0.94 and equaling 0.96 for the total score [31]. In present study, the Cronbach’s alpha for the subscales were 0.90 to 0.94 and equaling 0.96 for the total score.

### 2.7. Sociodemographic Characteristics

Age, gender, education level, marital status, and employment were self-reported by participants. Disease-related variables were collected from the medical records of participants such as comorbidities, weight, Body mass index (BMI), surgery type, and laboratory parameters etc. (Table 1).

### 2.8. Statistical Analysis

All statistical analyses were performed using R software (version 4.4.1 for Windows). Data Description and Imputation Cohort characteristics were summarized using descriptive statistics (median and interquartile range, mean and standard deviation, frequency, numbers and percentages). Variable correlations were explored via a correlation coefficients matrix. Missing data were addressed using multiple imputation tailored to variable types: Predictive Mean Matching (PMM) for numerical variables, logistic regression for binary factors, and polynomial regression for multi-classification factors. Modeling and Trajectory Analysis Group-Based Trajectory Modeling (GBTM) was applied to analyze one-year post-bariatric surgery trajectories of eating behavior and HRQoL in 244 patients. Trajectory models were estimated using all scheduled postoperative assessment time points, including 1 week (baseline), 1 month, 3 months, 6 months, 9 months, and 12 months. Model fit was determined by the Bayesian Information Criterion (BIC), requiring a minimum 5% sample inclusion per trajectory. A linear multiple regression model was subsequently used to assess the impact of these eating behavior trajectories on post-surgical QoL. To account for the varying metabolic and restrictive impacts of different procedures, surgery type (laparoscopic sleeve gastrectomy (LSG), one anastomosis gastric bypass (OAGB), and single anastomosis duodeno–ileal by-pass with sleeve gastrectomy (SADI-S)) was explicitly included as a categorical control variable in all regression models, alongside other potential confounders such as age, gender, and baseline BMI. Two-sided tests were performed, and the significance level was set at 0.05 (*p* < 0.05).

## 3. Results

### 3.1. The Characteristics of Participants

**Figure 1:**
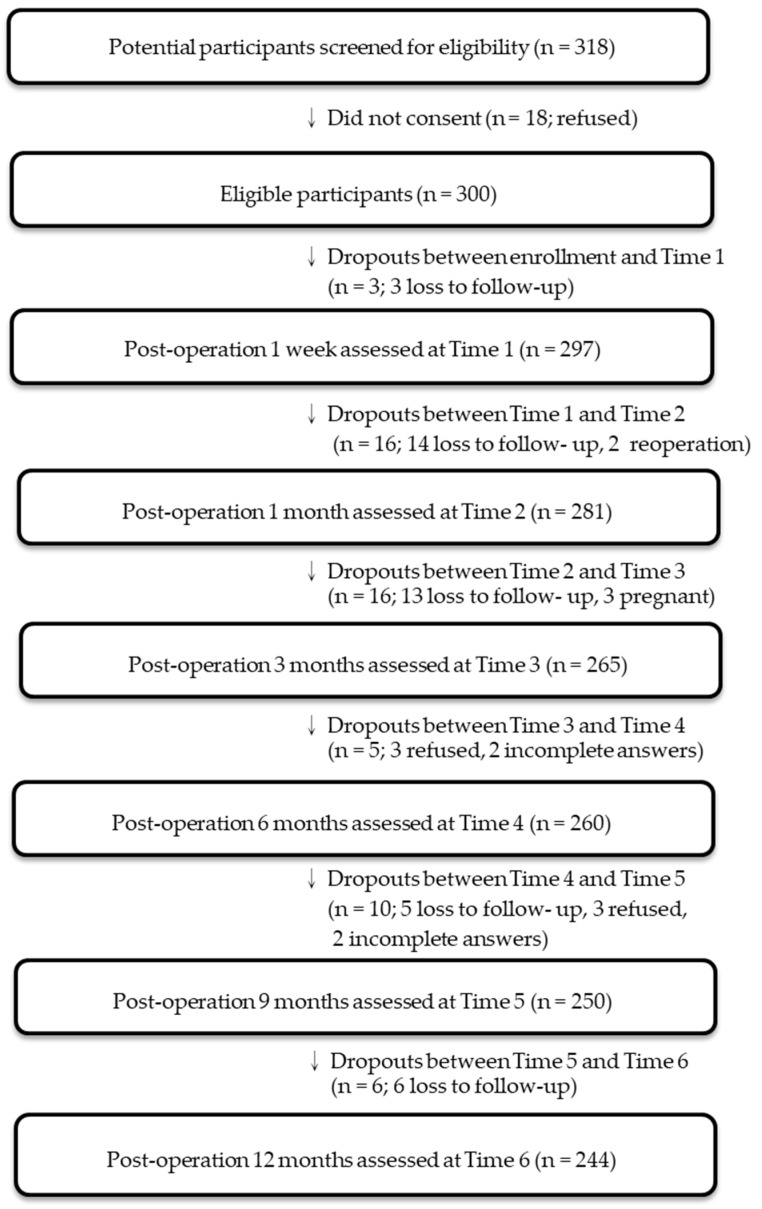
Flow diagram of participants follow-up. Arrows indicate progression to the next step in the study process.

Of the 300 participants initially enrolled, 3 withdrew before the first follow-up, 16 before the second, 16 before the third, 5 before the fourth, 10 before the fifth, and 6 before the sixth. Ultimately, 244 participants completed all six follow-ups and were included in the final analysis (Figure 1). Among the 244 participants, 65.2% (*n* = 159) were female, with a mean age of 36.1 years (SD = 9.05; range: 20–66). Most participants were unmarried (59.8%, *n* = 146), had a senior high school education or below (52.1%, *n* = 127), and were unemployed (50.4%, *n* = 123). The mean postoperative body weight was 102.4 ± 19.3 kg (range: 56.5–169.2), and the mean BMI was 37.1 ± 5.6 kg/m^2^ (range: 24.9–59.1).

Most participants had at least one obesity-related comorbidity (91.8%, *n* = 224) and underwent metabolic bariatric surgery. Regarding surgical procedures, 38.9% (*n* = 95) received laparoscopic sleeve gastrectomy (LSG), 34.8% (*n* = 85) underwent one anastomosis gastric bypass (OAGB), and 26.2% (*n* = 60) received single anastomosis duodeno–ileal bypass with sleeve gastrectomy (SADI-S). Mean scores for restrained eating, emotional eating, external eating, and IWQOL-Lite increased or decreased from baseline to 12 months postoperatively, ranging from 26.8 ± 5.9 to 28.4 ± 7.2, 33.4 ± 11.9 to 28.3 ± 9.1, 31.3 ± 5.9 to 26.1 ± 5.9, and 58.2 ± 19.8 to 83.9 ± 16.2, respectively. Detailed baseline sociodemographic and clinical characteristics are presented in Table 1.

### 3.2. Trajectories of Postoperative Eating Behavior and Across Three Domains

**Figure 2:**
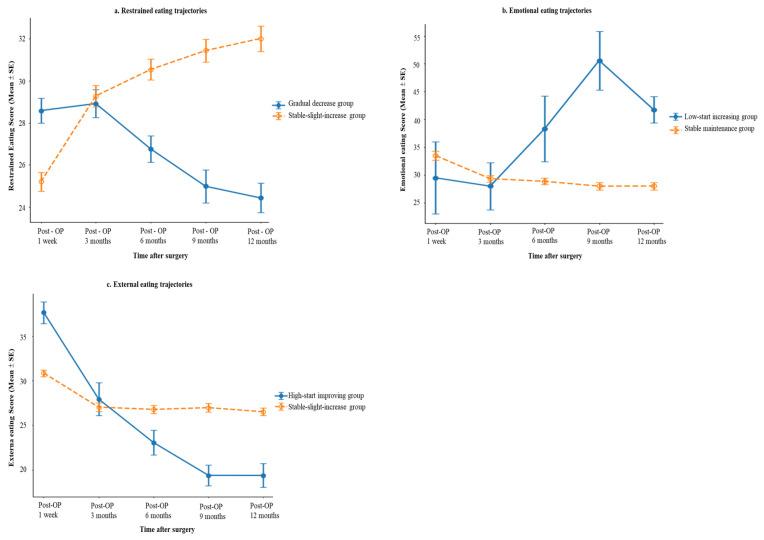
Eating Behavior Trajectories. (**a**) Restrained eating trajectory; (**b**) Emotional eating trajectory; (**c**) External eating trajectory.

GBTM indicated that two-group models provided the best fit for restrained eating (BIC = 2702.76), emotional eating (BIC = 2518.82), and external eating (BIC = 2658.28) (Figure 2).

For restrained eating, two trajectories were identified (Figure 2a): a gradual decrease group (48.0%, *n* = 117), characterized by a gradual decline over time (β = −0.056, *p* = 0.042), and a stable-slight-increase group (52.0%, *n* = 127), which showed a modest upward trend (β = 0.053, *p* = 0.033). For emotional eating (Figure 2b), the majority of participants belonged to a stable maintenance group (97.5%, *n* = 238), while a small subgroup followed a low-start increasing group (2.5%, *n* = 6; β = 0.193, *p* < 0.001). For external eating (Figure 2c), most participants were classified into a stable-slight-increase group (92.6%, *n* = 226), whereas a smaller subgroup exhibited a high-start improving group (7.4%, *n* = 18; β = −0.153, *p* < 0.001). Trajectory details are shown in Table 2.

### 3.3. Trajectories of Postoperative HRQoL and Across Five Domains

**Figure 3:**
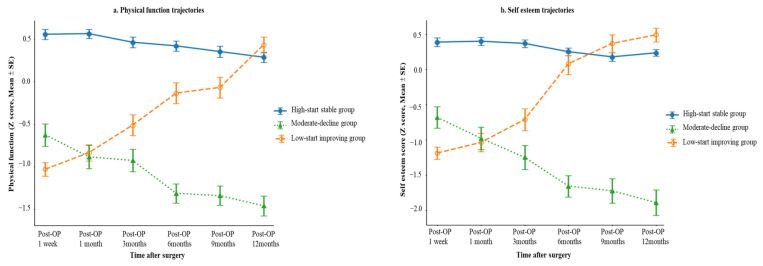

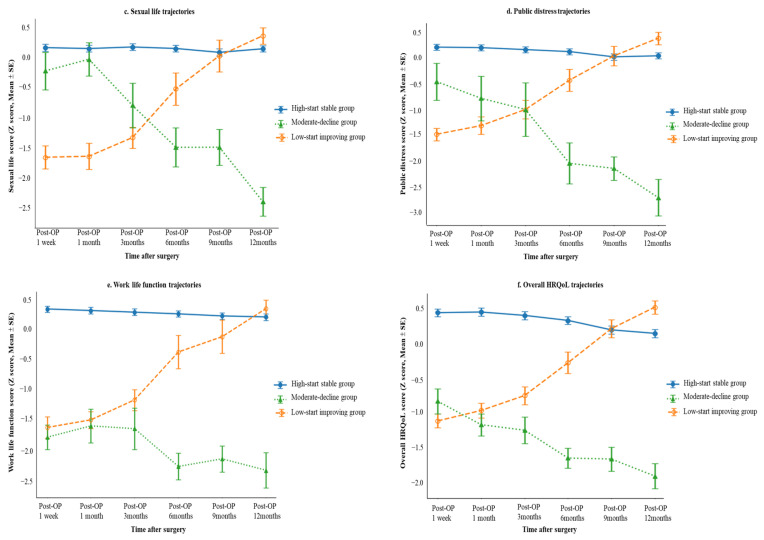
Health-related Quality of Life Trajectories. (**a**) Physical function trajectory; (**b**) Self-esteem trajectory; (**c**) Sexual life trajectory; (**d**) Public distress trajectory; (**e**) Work life function trajectory; (**f**) overall HRQoL trajectory.

Across HRQoL domains, three-group trajectory models provided the best balance between statistical model fit and clinical interpretability (Figure 3). Consistently across physical function, work life function, self-esteem, sexual life, public distress, and overall HRQoL, approximately 45–50% of patients followed a high-start stable trajectory, 30–35% a moderate-decline trajectory, and about 20% a low-start improving trajectory during the first postoperative year. The high-start stable group maintained relatively favorable functioning across domains with only minor declines over time. In contrast, the low-start improving group demonstrated marked and sustained postoperative improvements, particularly after the mid-postoperative period, with scores approaching or surpassing baseline levels by 12 months.

Notably, a clear contrast was observed between the low-start improving group and the moderate-decline group. Although the low-start improving group presented with the poorest baseline HRQoL, these patients exhibited substantial recovery across all domains over time. Conversely, the moderate-decline group, despite having relatively higher baseline HRQoL, showed progressive deterioration across physical, work life, and psychosocial domains. This divergence highlights the heterogeneous nature of postoperative recovery and suggests that baseline HRQoL alone may not adequately predict longitudinal outcomes. The moderate-decline group, in particular, may represent a clinically vulnerable subgroup at risk for persistent impairment despite surgery. Overall HRQoL trajectories closely mirrored these domain-specific patterns, underscoring substantial heterogeneity in postoperative quality-of-life adaptation. Detailed trajectory distributions and fit indices are presented in Table 2.

### 3.4. Associations of Sociodemographic, Clinical Factors, and Eating Behavior Trajectories with Health-Related Quality of Life

The results of the multivariate analysis, including Baseline BMI, sex, age, marital status, educational level, occupation type, smoking and alcohol consumption, surgery type, and other clinically relevant variables as a control variable, are now summarized in Table 3. The overall model was statistically significant (F (48, 195) = 2.34, *p* < 0.001), explaining 37% of the variance in HRQoL (R^2^ = 0.37; adjusted R^2^ = 0.21). The intercept was estimated at 123.69, representing the estimated HRQoL score when all independent variables were set at their reference categories.

Among the variables included in the model, higher BMI (β = −1.17, *p* = 0.017), membership in the high depressive symptom stable trajectory group (β = −19.56, *p* = 0.001), and asthma comorbidity (β = −23.89, *p* = 0.011) were significantly associated with lower HRQoL at 12 months. In contrast, higher PTH levels were positively associated with HRQoL (β = 0.09, *p* = 0.020). These findings indicate that psychological and clinical factors contributed more substantially to the explained variance in HRQoL than eating behavior trajectories during the first postoperative year.

In terms of the effects of eating behavior trajectory changes, the analysis of re-strained eating showed that, compared with the reference group (“stable slight in-crease”), the “gradual decrease” group had a coefficient of −1.42 (*p* = 0.502, 95% CI = −5.57 to 2.74), which was not statistically significant. For emotional eating, the results indicated that, compared with the reference group (“low-start increasing”), the “stable maintenance” group had a coefficient of −10.33 (*p* = 0.110, 95% CI = −23.03 to 2.37). Although not statistically significant, this finding approached significance and suggests that a stable pattern of emotional eating may be associated with poorer quality of life. In the analysis of external eating, compared with the reference group (“high-start im-proving”), the “stable slight increase” group had a coefficient of −5.33 (*p* = 0.160, 95% CI = −12.79 to 2.13), which did not reach statistical significance.

## 4. Discussion

The present study investigated the longitudinal trajectories of eating behaviors and HRQoL among patients undergoing MBS. Consistent with previous literature, significant improvements in weight and HRQoL were observed during the first postoperative year [4,5,20,21,22,24]. However, these average improvements masked substantial inter-individual variability. Rather than a uniform recovery pattern, distinct behavioral and psychosocial adaptation trajectories emerged. Such heterogeneity is increasingly recognized in bariatric research, where individuals with comparable weight loss may experience markedly divergent psychosocial outcomes [10,20,32].

A methodological strength of the present study was the use of Group-Based Trajectory Modeling (GBTM), which enabled identification of latent subgroups that may be obscured in conventional aggregate analyses. This person-centered approach facilitates recognition of clinically meaningful recovery patterns and supports more precise risk stratification during postoperative follow-up [8]. Across HRQoL domains, three consistent trajectories were identified: high-start stable, low-start improving, and moderate-decline. Notably, nearly one-third of patients followed a moderate-decline pattern despite undergoing surgery. This finding has important clinical and public health implications, as it suggests that a substantial subgroup of patients may not experience sustained improvements in quality of life despite successful weight loss. These results underscore the importance of monitoring psychosocial outcomes in addition to weight-based indicators and support early identification of patients at risk of declining HRQoL [33,34].

A particularly notable contrast was observed between the low-start improving group and the moderate-decline group. Although patients in the low-start improving group (~20%) reported the poorest baseline HRQoL, they demonstrated substantial and sustained improvements throughout the first postoperative year. This pattern may reflect greater perceived benefit and enhanced psychological adaptation following marked weight loss. Previous studies similarly suggest that individuals with poorer baseline HRQoL or greater clinical burden often experience larger relative gains after MBS [35,36]. In contrast, patients in the moderate-decline group (~30–35%), despite having relatively higher baseline HRQoL, exhibited progressive deterioration over time. This pattern suggests that baseline HRQoL alone may not adequately predict postoperative trajectories. Recent trajectory studies have likewise demonstrated subgroups with declining or suboptimal HRQoL associated with psychological and clinical vulnerabilities [11,33].

From a clinical perspective, these findings highlight the importance of early identification and targeted support for patients at risk of unfavorable HRQoL trajectories. Potential interventions may include preoperative education to promote realistic expectations, postoperative psychological counseling, routine screening for depressive symptoms, and strategies to strengthen coping skills and emotional regulation. A multidisciplinary follow-up model integrating psychological support with standard medical care may therefore be important for optimizing long-term outcomes.

Parallel heterogeneity was observed in eating behaviors. Restrained, emotional, and external eating followed distinct postoperative patterns, underscoring the complexity of behavioral adaptation after MBS. Previous studies have linked maladaptive eating behaviors to poorer weight-loss and psychosocial outcomes [15,32]. The trajectory-based findings further demonstrate that eating patterns evolve dynamically and may contribute to the variability in HRQoL outcomes, though the present study design precludes causal inferences.

From a behavioral regulation perspective, postoperative restrained eating may reflect adaptive self-management rather than pathological restriction. Sustained adherence to dietary guidelines and consistent self-monitoring are essential for long-term surgical success [9,32]. Individuals who maintain appropriate restraint while reducing emotional and external eating may gain a sense of perceived control, potentially bolstering psychosocial health. Conversely, persistent emotional eating may signal ongoing difficulties in affect regulation. Emotional eating remains a key indicator of psychological vulnerability and poorer postoperative adjustment [15,37]. When food continues to serve as a primary coping mechanism, weight reduction alone may fail to yield corresponding improvements in perceived well-being.

Expectation experience discrepancy may further help explain the moderate-decline trajectory. Bariatric candidates often hold high expectations regarding improvements in appearance, social functioning, and overall well-being [34]. When postoperative outcomes fail to meet these expectations, quality of life may decline despite objective clinical improvement. This mismatch has been associated with dissatisfaction and psychological distress [34,38]. Furthermore, substantial weight loss can alter patients’ identity, interpersonal relationships, and occupational roles. While many individuals adapt successfully to these changes, others may experience role strain or adjustment difficulties [10,34]. In this context, factors such as weight stigma and evolving social interactions may further influence psychological outcomes [19]. Therefore, early identification of such discrepancies may allow timely intervention through expectation management, cognitive-behavioral therapy, and supportive counseling.

Notably, although eating behavior trajectories were associated with domain-specific HRQoL, they were not independently associated with total HRQoL after adjustment for early postoperative BMI and comorbidities. Early weight status emerged as a key determinant of 12-month HRQoL, reinforcing evidence that early postoperative weight trajectories significantly influence patient satisfaction [20,24]. Several methodological and clinical considerations may help explain this finding. Although surgery type was included as a covariate in the regression model, different bariatric procedures may exert distinct effects on postoperative adaptation due to variations in restrictive, hormonal, and metabolic mechanisms. Previous studies have demonstrated that Roux-en-Y gastric bypass, one anastomosis gastric bypass, and sleeve gastrectomy differ in their long-term impacts on bariatric outcomes and health-related quality of life [6,7,39,40]. However, subgroup analyses by surgery type were not performed in the present study, which may have limited the ability to detect procedure-specific effects.

In addition, the regression model explained 37% of the variance in HRQoL; however, eating behavior trajectories were not identified as significant independent predictors. This result should be interpreted cautiously, as it may reflect overadjustment, multicollinearity, and limited statistical power for smaller trajectory subgroups. Accordingly, the independent contribution of eating behavior trajectories may have been attenuated within a multivariable framework containing strongly interrelated clinical and psychological factors [41,42], which may partly explain why clinical and psychological variables emerged as stronger predictors of HRQoL in the present model. Accordingly, clinical and psychological factors demonstrated stronger associations with HRQoL outcomes, suggesting that early postoperative quality of life may be more strongly driven by clinical burden and psychological status than by short-term behavioral changes. This interpretation is supported by recent evidence highlighting the central role of psychological variables such as depression, coping strategies, and body image in shaping postoperative outcomes [43,44].

In summary, these findings suggest that early postoperative HRQoL may be more strongly driven by clinical burden and psychological status than by short-term behavioral changes. During the first postoperative year, rapid physiological improvements such as weight loss and remission of obesity related comorbidities may exert a more immediate influence on overall quality of life, potentially obscuring the independent effects of eating behaviors. Importantly, this does not preclude the relevance of eating behaviors in the longer term. The impact of eating behavior patterns may become more pronounced over extended follow-up periods (e.g., 2–5 years), as behavioral habits stabilize and their cumulative influence on weight maintenance and psychosocial functioning emerges. This interpretation is supported by longitudinal evidence indicating that while HRQoL improvements are most prominent within the first 1–2 years after bariatric surgery, longer-term outcomes are increasingly influenced by behavioral and psychosocial factors [39]. Therefore, the absence of a significant association at 12 months should be interpreted with caution, and longer-term follow-up is essential to fully understand the temporal relationship between eating behavior trajectories and HRQoL outcomes.

### 4.1. Clinical Implications

These findings emphasize that postoperative progress after MBS should be evaluated using broader indicators than weight loss alone. Early identification of patients exhibiting persistent emotional eating or declining HRQoL allows for timely psychosocial interventions [9,13,37]. A structured, multidisciplinary follow-up model integrating nutritional counseling, expectation management, and psychological support is essential to optimizing long-term outcomes.

### 4.2. Limitations

Several limitations warrant consideration. First, the use of convenience sampling from two hospitals in Southern Taiwan may limit the generalizability of the findings. This study was conducted within a specific cultural and healthcare context, where dietary patterns, family support systems, cultural attitudes toward obesity, and access to postoperative follow-up care may differ from those in other regions. Such contextual factors may influence eating behavior adaptation, psychological adjustment, and HRQoL trajectories after metabolic and bariatric surgery. Accordingly, although the overall pattern of heterogeneous postoperative recovery may be relevant to other bariatric populations, the prevalence, magnitude, and composition of specific trajectory groups may vary across ethnic, cultural, and healthcare settings. Therefore, caution is warranted when generalizing these findings to populations with different sociocultural or clinical care backgrounds, and external validation in diverse populations is needed. Second, the one-year follow-up period only captures short-to-medium-term outcomes; longer-term studies are critical, as weight regain and behavioral relapse often emerge beyond the first year. Third, certain psychological variables, such as depression, anxiety, and social support, were not comprehensively analyzed. Fourth, the lack of a structured psychological management program, while reflecting local standard care, may limit the applicability of findings to healthcare systems with mandatory multidisciplinary follow-up. However, these results provide a valuable baseline for comparing natural postoperative trajectories with those observed under structured intervention frameworks. In this context, the absence of systematic psychological support may also have contributed to the observed heterogeneity in HRQoL trajectories and the limited independent effect of behavioral factors at 12 months. Fifth, the relatively small size of certain trajectory subgroups may reduce the robustness of subgroup-specific estimates. In addition, the number of predictors included in the regression analyses relative to the available sample size may have reduced statistical power and increased uncertainty around the estimated effects. Accordingly, non-significant findings should be interpreted cautiously, as the study may have been underpowered to detect small-to-moderate associations. Future research should employ multicenter and cross-cultural samples, extend follow-up beyond the first postoperative year, and apply advanced longitudinal methods such as structural equation modeling or mixed-methods approaches to better clarify the causal pathways linking eating behavior, psychological adaptation, and HRQoL. Qualitative studies may also provide deeper insight into the lived experiences of postoperative patients.

## 5. Conclusions

Postoperative adaptation following MBS is a highly heterogeneous process that transcends weight loss. The distinct trajectories identified in HRQoL and eating behaviors highlight that surgical success is multifaceted. While early postoperative BMI and comorbidities are primary drivers of overall QoL, eating behavior trajectories significantly influence specific psychosocial domains. These findings support a biopsychosocial framework for postoperative care and suggest that weight reduction alone may not fully capture patient recovery. Routine follow-up should therefore incorporate assessment of psychological well-being, eating behavior adaptation, and quality of life in addition to traditional weight-based outcomes. Early identification of patients at risk for unfavorable trajectories may facilitate timely multidisciplinary intervention and improve longer-term outcomes. Overall, personalized and integrated postoperative management may be essential to sustain the full long-term benefits of bariatric surgery.

## Figures and Tables

**Table 1:** Sociodemographic and Disease-related Characteristics of the Participants.

Variables	*n* (%)/Mean ± SD
Gender	
Female	159 (65.2)
Men	85 (34.8)
Age (years)	36.1 ± 9.1
Education level	
Senior high school or below	127 (52.1)
University or above	117 (47.9)
Marital status	
Unmarried/Divorced/Widowed	146 (59.8)
Married/partnered	98 (40.2)
Employment status	
Unemployed	123 (50.4)
Employed	121 (49.6)
Smoking	
No	189 (77.5)
Yes	55 (22.5)
Drinking	
No	221 (90.6)
Yes	23 (9.4)
Weight	
Preoperative	108.6 ± 20.4
1 week after operation	102.4 ± 19.3
12 months after surgery	72.3 ± 13.3
Body mass index (BMI)	
Preoperative	39.4 ± 5.9
1 week after operation	37.1 ± 5.6
12 months after surgery	26.5 ± 3.9
Comorbidities	
No	20 (8.2)
Yes	224 (91.8)
Surgery type	
LSG	95 (38.9)
OAGB	85 (34.9)
SADI-S	60 (26.2)
Restrained eating behavior score	
1 week after surgery	26.8 ± 5.9
3 months after surgery	29.1 ± 6.4
6 months after surgery	28.7 ± 6.5
9 months after surgery	28.2 ± 7.0
12 months after surgery	28.4 ± 7.2
Emotional eating behavior score	
1 week after surgery	33.4 ± 11.9
3 months after surgery	29.3 ± 9.1
6 months after surgery	29.1 ± 9.2
9 months after surgery	28.7 ± 9.8
12 months after surgery	28.3 ± 9.1
External eating behavior score	
1 week after surgery	31.3 ± 5.9
3 months after surgery	27.1 ± 7.5
6 months after surgery	26.5 ± 6.5
9 months after surgery	26.3 ± 6.5
12 months after surgery	26.1 ± 5.9
IWQOL-Lite score	
1 week after surgery	58.2 ± 19.8
1 month after surgery	67.8 ± 19.2
3 months after surgery	73.4 ± 17.3
6 months after surgery	78.2 ± 17.2
9 months after surgery	82.8 ± 16.2
12 months after surgery	83.9 ± 16.2

**Table 2:** Trajectory groups of eating behaviors and HRQoL identified by group-based modeling (N = 244).

Trajectory Group	*n* (%)	Baseline β (*p*)	Slope β (*p*)	Description
Restrained Eating				
Gradual decrease group	117 (48.0)	0.20 (0.272)	−0.06 (0.042)	Higher initial level; gradual decline
Stable-slight-increase group	127 (52.0)	−0.18 (0.097)	0.05 (0.033)	Lower initial level; gradual increase
Emotional Eating				
Low-start increasing group	6(2.5)	−0.52 (0.297)	0.19 (<0.001)	Low baseline; significant upward trend
Stable maintenance group	238 (97.5)	0.02 (0.793)	−0.01 (0.395)	Stable; no significant change
External Eating				
Stable-slight-increase group	226 (92.6)	−0.07 (0.315)	0.02 (0.013)	Slightly lower baseline; mild upward trend
High-start improving group	18 (7.4)	0.57 (0.115)	−0.15 (<0.001)	Higher baseline; significant decline
Physical function domain				
High-start stable	122 (50.0)	0.53 (<0.001)	−0.02 (0.009)	High baseline; minimal decline
Moderate-decline	73 (29.9)	−0.58 (<0.001)	−0.06 (0.002)	Lower baseline; gradual decline
Low-start improving	49 (20.1)	−0.83 (<0.001)	0.10 (<0.001)	Lowest baseline; significant improvement
Self-esteem domain				
High-start stable	110 (45.1)	0.41 (<0.001)	−0.02 (0.011)	High baseline, stable
Moderate-decline	85 (34.8)	−0.82 (<0.001)	−0.10 (<0.001)	Lower baseline, declining
Low-start improving	49 (20.1)	−1.00 (<0.001)	0.14 (<0.001)	Lowest baseline, improving
Sexual life domain				
High-start stable	117 (48.0)	0.17 (0.004)	−0.004 (0.524)	High baseline, stable
Moderate-decline	78 (31.9)	−0.03 (0.911)	−0.20 (<0.001)	Average baseline, significant decline
Low-start improving	49 (20.1)	−1.56 (<0.001)	0.17 (<0.001)	Lowest baseline, improving
Public distress domain				
High-start stable	115 (47.1)	0.24 (<0.001)	−0.01 (0.012)	High baseline, stable
Moderate-decline	80 (32.8)	−0.46 (0.111)	−0.18 (<0.001)	Lower baseline, declining
Low-start improving	49 (20.1)	−1.30 (<0.001)	0.15 (<0.001)	Lowest baseline, improving
Work life function domain				
High-start stable	110 (45.1)	0.28 (<0.001)	−0.01 (0.038)	High baseline, stable
Moderate-decline	85 (34.8)	−1.50 (<0.001)	−0.07 (0.021)	Low baseline, gradual decline
Low-start improving	49 (20.1)	−1.48 (<0.001)	0.16 (<0.001)	Low baseline, improving
Overall HRQoL				
High-start stable	112 (45.9)	0.45 (<0.001)	−0.03 (<0.001)	High baseline, stable
Moderate-decline	83 (34.0)	−0.89 (<0.001)	−0.08 (<0.001)	Lower baseline, declining
Low-start improving	49 (20.1)	−1.04 (<0.001)	0.13 (<0.001)	Lowest baseline, improving

β = standardized regression coefficient; *p* = *p*-value. Percentages and sample sizes represent group membership.

**Table 3:** Multiple regression analysis of factors associated with HRQoL at 12 months after metabolic bariatric surgery (N = 244).

Variable	β	SE	t	*p*	95% CI
Intercept	126.69	26.53	4.66	<0.001	71.38, 176.01
Age	0.06	0.16	0.37	0.715	0.25, 0.37
Sex (Reference: Female)					
Men	−1.42	3.85	−0.37	0.713	−9.01, 6.18
Baseline BMI	−1.17	0.49	−2.4	0.017 *	−2.13, −0.21
Surgery Type (Referencef: LSG)					
OAGB	3.36	5.44	0.62	0.537	−7.36, 14.09
SADI-S	−3.49	2.62	−1.33	0.184	−8.66, 1.68
Asthma	−23.89	9.33	−2.56	0.011 *	−42.29, −5.49
PTH levels	0.09	0.04	2.35	0.020 *	0.01, 0.17
High Depressive Symptom (Stable)	−19.56	5.82	−3.36	0.001 *	−31.04, −8.08
Restrained Eating Trajectory (Reference: Stable-slight-increase group)					
High-start Improving	−1.42	2.11	−0.67	0.502	−5.57, 2.74
Emotional Eating Trajectory (Reference: Low-start increasing group)					
Stable Maintenance	−10.33	6.45	−1.6	0.11	−23.03, 2.37
External Eating Trajectory (Reference: High-start improving group)					
Stable-slight-increase	−5.33	3.78	−1.41	0.16	−12.79, 2.13

Note. SE: mean ± standard error; LSG: laparoscopic sleeve gastrectomy; OAGB: one anastomosis gastric bypass; SADI-S: single anastomosis duodenal switch; * *p* < 0.05.

## Data Availability

The datasets generated and analyzed during the current study are not publicly available due to patient privacy concerns and ethical restrictions. However, they are available from the corresponding author upon reasonable request and subject to the completion of a data-sharing agreement.

## Abbreviations

The following abbreviations are used in this manuscript:

MBS	MBS Metabolic bariatric surgery
HRQoL	Health-related quality of life
QoL	Quality of life
GBTM	Group-based trajectory modeling
DEBQ	Dutch Eating Behavior Questionnaire
IWQOL-Lite	Impact of Weight on Quality of Life-Lite
BMI	Body Mass Index
LSG	Laparoscopic sleeve gastrectomy
OAGB	One anastomosis gastric bypass
SADI-S	Single anastomosis duodenal switch
BIC	Bayesian Information Criterion
SD	Standard Deviation
CI	Confidence Interval
